# A stochastic hybrid systems based framework for modeling dependent failure processes

**DOI:** 10.1371/journal.pone.0172680

**Published:** 2017-02-23

**Authors:** Mengfei Fan, Zhiguo Zeng, Enrico Zio, Rui Kang, Ying Chen

**Affiliations:** 1 School of Reliability and Systems Engineering, Beihang University, Beijing, China; 2 Chair System Science and the Energy Challenge, Fondation Electricité de France (EDF), CentraleSupélec, Université Paris Saclay, Chatenay-Malabry, France; 3 Energy Department, Politecnico di Milano, Milan, Italy; Southwest University, CHINA

## Abstract

In this paper, we develop a framework to model and analyze systems that are subject to dependent, competing degradation processes and random shocks. The degradation processes are described by stochastic differential equations, whereas transitions between the system discrete states are triggered by random shocks. The modeling is, then, based on Stochastic Hybrid Systems (SHS), whose state space is comprised of a continuous state determined by stochastic differential equations and a discrete state driven by stochastic transitions and reset maps. A set of differential equations are derived to characterize the conditional moments of the state variables. System reliability and its lower bounds are estimated from these conditional moments, using the First Order Second Moment (FOSM) method and Markov inequality, respectively. The developed framework is applied to model three dependent failure processes from literature and a comparison is made to Monte Carlo simulations. The results demonstrate that the developed framework is able to yield an accurate estimation of reliability with less computational costs compared to traditional Monte Carlo-based methods.

## Introduction

Failure of industrial components, systems and products may be caused by multiple failure processes, e.g. wear, corrosion, erosion, creep, fatigue, etc. [1]. In general, the failure processes are categorized as degradation processes (or soft failures) and catastrophic failure processes (or hard failures) [2]. Soft failure is caused by continuous degradation and is often modeled by a continuous-state random process, e.g., Wiener process [3,4], Gamma process [5–7], inverse Gaussian process [8–10], continuous-time semi Markov process [11], etc. Hard failure is caused by traumatic shocks in various patterns and is often modeled by a discrete-state random process, e.g., Homogeneous Poisson Process (HPP) [11–13], Nonhomogeneous Poisson Process (NHPP) [14–16], etc. Often, complex dependencies exist among the failure processes [17]. For example, [18] presents experimental data to show that erosion and corrosion can enhance each other and therefore accelerate the failure process. Also, it is observed in [19] that the dependency between creep and fatigue severely reduces the Time-To-Failure (TTF) of the specimens that are exposed to high temperatures and heavy loads. To accurately describe the failure behavior affected by multiple failure processes, the possible dependencies among the failure processes need to be properly addressed.

In literature, various methods have been developed to model the dependencies among degradation processes and random shocks. Peng et al. [20] develop a dependency model where the arrived shocks lead to an abrupt increase of the degradation process. Wang and Pham [21] investigate systems subject to dependent competing risk, which suffer failures due to degradations and random shocks: the model is proposed of shocks that can cause immediate failure of the system, with a time-dependent probability *p*(*t*), or can increase the degradation level with probability 1−*p*(*t*). Cha and Finklstein [22] assume that a shock can lead to a hard failure with probability *p*(*t*), or can increase the degradation rate with probability 1−*p*(*t*). Jiang et al. [23] develop a model that considers that the threshold of hard failures can be shifted by random shocks. Rafiee et al. [24] consider that the degradation rate is increased by a series of shocks. Jiang et al. [1] categorize shocks into different shock zones based on their magnitudes and consider that shocks in different zones have different effects on the degradation process. Bagdonavicius et al. [25], Fan et al. [26] and Ye et al. [27] develop models that consider that the probability of hard failures is increased as the degradation process progresses. Huynh et al. [14,15] investigate maintenance strategies for a dependence model, where the intensity of the NHPP for random shock is a piecewise function of the degradation magnitude. Fan et al. [16] present a reliability model for sliding spools considering that the intensity of the NHPP describing the random shock process is a linear function of the degradation level.

For models that consider the dependencies between degradation and random shock processes, like these above, it is often too complicated, if not intractable, to evaluate system reliability analytically. Then, simulation methods, such as Monte Carlo methods [28], are used, often with limitations due to heavy computational burden. In this respect, Stochastic Hybrid Systems (SHS) [29] offer a new way to model the stochastic behavior of systems that involve both discrete and continuous states [30–33]. SHS describe the system’s behavior by a set of differential equations and therefore, whose solution avoids the computational burdens of simulation methods. Various forms of SHS exist in literature (see Pola et al. [29] for a comparison). In this paper, we adopt the models recently developed by Hespanha in [34–36], which is similar to the Piecewise Deterministic Markov Process (PDMP) [37] but differs from it in that the continuous state variable follow Stochastic Differential Equations (SDEs), rather than Ordinary differential equations (ODEs). To the best of our knowledge, it is the first attempt to use SHS for modeling dependent failure processes.

It should be mentioned that SHS is similar to Stochastic Hybrid Automata (SHA), which is also applied in Dynamic Reliability (DR) assessment or Dynamic Probabilistic Risk Assessment (DPRA) [38,39]. Both methods model dynamic hybrid system behaviors that involve stochastic factors. SHA introduces less assumptions than SHS and resorts to Monte Carlo simulation for the calculations [40,41]; SHS, on the other hand, is able to describe the hybrid dynamics analytically or semi-analytically, by solving a set of Differential Equations (DEs) on the expense of introducing more assumptions [36]. The computational cost of SHS is, in general, less than that of SHA, but on the expense of more assumptions in particular with respect to the degradation models, whereby epistemic uncertainty (specifically model uncertainty) [42–44] is introduced. When applying the SHS in practice, then, care should be taken to ensure that the assumptions are consistent with the actual situation, in particular in case of systems characterized by complex and numerous dependencies among physical processes and failure behaviors. In this paper, we choose SHS because the type of dependent degradation and shock processes allows for modeling by SHS and, in general, SHS has a better computational performance than SHA.

## Methods

### SHS model

The state space of a SHS model is a combination of discrete and continuous states. Let us denote the discrete states by *q*(*t*), *q*(*t*) ∈*Q*, where *Q* is a finite set containing all the possible discrete modes of the system. The continuous states are denoted by *x*(*t*), *x*(*t*)∈ℝ^*l*^. A SHS model is defined based on the following assumptions [34–36]:

1. The evolution of the continuous states is governed by a set of SDEs:
  $$dx\left(t\right)=f\left(q\left(t\right),x\left(t\right)\right)dt+g\left(q\left(t\right),x\left(t\right)\right)dw_{t},$$ (1)
  where *w*_*t*_: ℝ^+^ → ℝ^*k*^ is a k-dimensional Wiener process; *f*: *Q*×ℝ^*l*^ → ℝ^*l*^ and *g*: *Q*×ℝ^*l*^ → ℝ^*l*×*k*^, respectively.
2. At any time *t*, if the system is in state (*q*(*t*), *x*(*t*)), it undergoes a transition with a rate *λ*_*ij*_(*q*(*t*), *x*(*t*)):*Q*×ℝ^*l*^ → ℝ^+^, *i*, *j* ∈*Q*. That is, the probability that the system undergoes a transition from state *i* to state *j* within the interval [*t*, *t* + Δ*t*) is:
  $$\lambda_{ij}\left(q\left(t\right),x\left(t\right)\right)\Delta t+o\left(\Delta t\right),$$ (2)
3. Whenever the system undergoes a state transition from state *i* to state *j*, it instantaneously applies the map *ϕ*_*ij*_(*q*(*t*), *x*(*t*)) to the current values of *q*(*t*) and *x*(*t*), so that their values are reset:
  $$\left(q\left(t\right),x\left(t\right)\right)=\phi_{ij}\left(q\left(t^{-}\right),x\left(t^{-}\right)\right),$$ (3)
  where the notation *a*(*t*^−^) represents the left-hand limit of the function *a* at time *t*.

**Fig 1:**
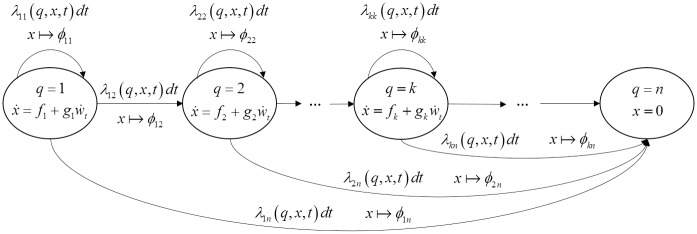
State-transition diagram for the SHS model.

Fig 1 depicts the state transition and evolution of the SHS.

### SHS formulism for dependent failure processes

The modeling framework for dependent failure processes involves three elements, i.e., a model for the degradation process, a model for the shock process and a model for the dependency between the two processes. The following assumptions are made in order to model a dependent failure process in the framework of SHS:

Assumption (1) The degradation processes are characterized by *x*(*t*) = (*x*_1_(*t*), *x*_2_(*t*), …, *x*_*l*_(*t*))∈ℝ^*l*^. The elements in *x*(*t*), *x*_*i*_(*t*), 1≤*i*≤*l*, are performance parameters for the degradation processes and are independent from one another. Soft failure occurs whenever
  $$\exists i\in \left\{1,2,\cdots ,l\right\},x_{i}\left(t\right)>H_{i},$$ (4)
  where *H*_*i*_ is the failure threshold for the performance parameter *x*_*i*_(*t*).
Assumption (2) The system has *n* potential health states, i.e., *q*(*t*)∈*Q* where *q*(*t*) is a discrete-state variable that quantifies the system’s health state at time *t*, and *Q* = {1, 2, …, *n*} is a set containing all the possible system states. When *q*(*t*) = *n*, a hard failure occurs.
Assumption (3) Transitions between system health states are triggered by the arrival of random shocks with the transition rate *λ*_*ij*_(*q*(*t*), *x*(*t*)), *i*, *j* ∈ *Q* where the probability that the system jumps from state *i* to state *j* in the interval [*t*, *t* + Δ*t*) is given by Eq (2).
Assumption (4) Between the transitions, the degradation of *x*(*t*) is characterized by the SDEs in Eq (1) for *q*(*t*) = 1, 2, ⋯, *n* − 1. When *q*(*t*) takes different values, the form of *f*(⋅) and *g*(⋅) can be changed to reflect the dependency behavior. When *q*(*t*) = *n*, which indicates that the system fails due to hard failure, we impose that *x*(*t*) = 0.
Assumption (5) An arrival random shock resets the current values of *q*(*t*) and *x*(*t*), using the reset map defined in Eq (3).
Assumption (6) System failure is caused by both soft and hard failures, whichever occurs first.

Given a dependent failure process, the following steps show how to model it in the framework of SHS:

Step 1: Modeling degradation. In this step, the performance parameters *x*(*t*) are identified to characterize the degradation processes. For the performance parameters, the SDEs in Eq (1) are developed to describe their degradation, considering both deterministic and stochastic characteristics. The deterministic characteristics are often described based on the physical knowledge on the degradation processes (e.g., using the Physics-of-Failure (PoF) models [19]), while the stochastic characteristics are modeled by a Wiener process, as shown in Eq (1).
Step 2: Modeling random shocks. In SHS, random shocks are considered as transitions among the system health states. The transition rates, *λ*_*ij*_(*q*(*t*), *x*(*t*)), *i*, *j* ∈ *Q*, need to be determined based on historical data or expert judgments.
Step 3: Modeling dependencies. Finally, the dependencies between the degradation processes and random shocks need to be considered. The dependencies can be modeled in various ways in SHS. For instance, by resetting the values for *x*(*t*), the reset map in Eq (3) can capture the influence of the random shock on the degradation process. Further, the functions *f*, *g* and even *λ* itself, as shown in Fig 1, are dependent on the current values of *x*(*t*) and *q*(*t*), which provides a versatile way to model the dependencies.

Note that in order to make sure that the developed SHS model is solvable in case that truncations techniques [36] are needed, for example Case 3 in this paper, the *f*_*i*_, *g*_*i*_, *λ*_*ij*_, *ϕ*_*ij*_, *i*, *j* ∈ *Q*. in the SHS model have to be polynomial functions of *x*(*t*).

### Conditional moments estimation

In this section, we derive the conditional expectations for the continuous state variables, i.e., $E\left[x_{j}^{p}\left(t\right)|q\left(t\right)=i\right],\;p\in \mathbb{N},i\in Q,j=1,2,\cdots ,l$, where *x*_*j*_(*t*) represent the *j*th element of *x*(*t*). The conditional expectations will be used in the next section for reliability analysis. Let us define a test function to be

$$\psi_{i}^{\left(m\right)}\left(q,x\right)=\{\begin{array}{ll} x^{m} & q=i\\ 0 & q\neq i \end{array},$$ (5)

where *m*: = (*m*_1_, *m*_2_, …, *m*_*l*_), *m*∈ℕ^*l*^, and $x^{m}:=x_{1}^{m_{1}}x_{2}^{m_{2}}\cdot \cdot \cdot x_{l}^{m_{l}}$, and let the *m*-order conditional moment of the continuous state *x* be

$$\begin{array}{ll} \mu_{i}^{\left(m\right)}\left(t\right) & ≔E\left[\psi_{i}^{\left(m\right)}\left(q,x\right)\right]\\ & =E\left[x^{m}\left(t\right)|q\left(t\right)=i\right]\cdot \text{Pr}\left\{q\left(t\right)=i\right\}. \end{array}$$ (6)

For a general test function *ψ*(*q*(*t*), *x*(*t*)), *ψ*: *Q*×ℝ^*l*^ →ℝ, which is twice continuously differentiable with respect to *x*, the evolution of its expected value is governed by Dynkin’s formula [36]:

$$\frac{dE\left[\psi \left(q\left(t\right),x\left(t\right)\right)\right]}{dt}=E\left[\left(L\psi \right)\left(q\left(t\right),x\left(t\right)\right)\right],$$ (7)

where (*Lψ*)(*q*, *x*) is the extended generator of SHS and ∀(*q*, *x*)∈*Q*×ℝ^*l*^, (*Lψ*)(*q*, *x*) is given by

$$\begin{array}{l} \left(L\psi \right)\left(q,x\right):=\frac{\partial \psi \left(q,x\right)}{\partial x}f\left(q,x\right)\\ +\frac{1}{2}trace\left(\frac{\partial^{2}\psi \left(q,x\right)}{\partial x^{2}}g\left(q,x\right)g{\left(q,x\right)}^{\prime }\right)\\ +\sum_{i,j\in Q}\lambda_{ij}\left(q,x\right)\left(\psi \left(\phi_{ij}\left(q,x\right)\right)-\psi \left(q,x\right)\right), \end{array}$$ (8)

where *∂ψ / ∂x* and *∂*^2^*ψ / ∂x*^2^ denote the gradient and Hessian matrix of *ψ*(*q*,*x*) with respect to *x*, respectively; *trace*(*A*) is the trace of the matrix *A*, i.e., the sum of elements on its main diagonal.

Substituting Eq (5) into Eq (7), we get a group of differential equations with respect to $\mu_{i}^{\left(m\right)}\left(t\right),i\in Q,m\in {\mathbb{N}}^{l}$:

$$d\mu_{i}^{\left(m\right)}\left(t\right)=E\left[L\left(\psi_{i}^{\left(m\right)}\right)\left(q\left(t\right),x\left(t\right)\right)\right]\cdot dt.$$ (9)

The evolution of $\mu_{i}^{\left(m\right)}\left(t\right)$ can be depicted by solving Eq (9). The conditional moments can, then, be obtained by assigning proper values for *m*: if we let *m* = (0, 0, …, 0) we have

$$\mu_{i}^{\left(0,0,\ldots ,0\right)}\left(t\right)=\text{Pr}\left\{q\left(t\right)=i\right\},i\in Q.$$ (10)

If we let

$$m=\left[m_{1},m_{2},\cdots ,m_{l}\right]:\{\begin{array}{cc} m_{j}=p, & \text{if }j=k,k\in \left\{1,2,\cdots ,l\right\},\\ m_{j}=0, & \text{if }j\neq k, \end{array}$$

where *m*_*j*_ denotes the *j*th element in *m* and *p* is a natural number, we have

$$\mu_{i}^{\left(m\right)}\left(t\right)=E\left[x_{k}^{p}\left(t\right)|q\left(t\right)=i\right]\cdot \text{Pr}\left\{q\left(t\right)=i\right\},i\in Q.$$ (11)

The conditional expectations, $E\left[x_{j}^{p}\left(t\right)|q\left(t\right)=i\right],\;p\in \mathbb{N},i\in Q,j=1,2,\cdots ,l$, can, then, be calculated by combining Eqs (10) and (11).

### Reliability analysis

From Assumption 6, system reliability can be expressed as:

$$R\left(t\right)=\text{Pr}\left(q\left(t\right)<n,x_{1}\left(t\right)<H_{1},x_{2}\left(t\right)<H_{2},\cdots ,x_{l}\left(t\right)<H_{l}\right).$$ (12)

From the law of total probability, we have

$$\begin{array}{ll} R\left(t\right) & =\text{Pr}\left(q\left(t\right)<n,x_{1}\left(t\right)<H_{1},x_{2}\left(t\right)<H_{2},\cdots ,x_{l}\left(t\right)<H_{l}\right)\\ & =\sum_{i=1}^{n-1}\text{Pr}\left(q\left(t\right)=i\right)\cdot \text{Pr}\left(x_{1}\left(t\right)<H_{1},x_{2}\left(t\right)<H_{2},\cdots ,x_{l}\left(t\right)<H_{l}|q\left(t\right)=i\right). \end{array}$$ (13)

Since we assume that the degradation processes are independent from one another, Eq (13) becomes

$$R\left(t\right)=\sum_{i=1}^{n-1}\left(\prod_{j=1}^{l}\text{Pr}\left(x_{j}\left(t\right)<H_{j}|q\left(t\right)=i\right)\right)\cdot \text{Pr}\left(q\left(t\right)=i\right)$$ (14)

In Eq (14), Pr(*q*(*t*) = *i*) can be calculated by Eq (10), Pr(*x*_*j*_(*t*) < *H*_*j*_|*q*(*t*) = *i*), *i* = 1, 2, ⋯, *n* − 1, *j* = 1, 2, ⋯, *l* can, instead, be approximated using the First Order Second Moment (FOSM) method [45], since we have the conditional moments for *x*_*j*_(*t*) Let $\mu_{x_{j}|q=i}\left(t\right)$ and $\sigma_{x_{j}|q=i}\left(t\right)$ denote the expected value and standard deviation of the random variable *x*_*j*_(*t*) conditioned on *q* = *i*, respectively. Then, $\mu_{x_{j}|q=i}\left(t\right)$ and $\sigma_{x_{j}|q=i}\left(t\right)$ can be calculated by

$$\begin{array}{ll} {\hat{\mu }}_{x_{j}|q=i}\left(t\right) & =E\left[x_{j}\left(t\right)|q\left(t\right)=i\right]=\frac{\mu_{i}^{\left(m^{*,j}\right)}\left(t\right)}{\text{Pr}\left(q\left(t\right)=i\right)}=\frac{\mu_{i}^{\left(m^{*,j}\right)}\left(t\right)}{\mu_{i}^{\left(0,0,\ldots ,0\right)}\left(t\right)},\\ {\hat{\sigma }}_{x_{j}|q=i}\left(t\right) & =\sqrt{E\left(x_{j}{\left(t\right)}^{2}|q\left(t\right)=i\right)-{\left(E\left(x_{j}\left(t\right)|q\left(t\right)=i\right)\right)}^{2}}\\ & =\sqrt{\frac{\mu_{i}^{\left(m^{**,j}\right)}\left(t\right)}{\mu_{i}^{\left(0,0,\ldots ,0\right)}\left(t\right)}-{\left(\frac{\mu_{i}^{\left(m^{*,j}\right)}\left(t\right)}{\mu_{i}^{\left(0,0,\ldots ,0\right)}\left(t\right)}\right)}^{2}},i\in \left\{1,2,\ldots ,n-1\right\}, \end{array}$$ (15)

where m*^,*j*^ and m**^,*j*^ are given by

$$\begin{array}{ll} m^{*,j} & =\left[m_{1},m_{2},\cdots ,m_{l}\right]:m_{k}=1,\text{ if }k=j;m_{k}=0,\text{ if }k\neq j,\\ m^{**,j} & =\left[m_{1},m_{2},\cdots ,m_{l}\right]:m_{k}=2,\text{ if }k=j;m_{k}=0,\text{ if }k\neq j. \end{array}$$ (16)

Based on FOSM [45], Pr(*x*_*j*_(*t*) < *H*_*j*_|*q*(*t*) = *i*) can be approximated by

$$\text{Pr}\left(x_{j}\left(t\right)<H_{j}|q\left(t\right)=i\right)\approx \Phi \left(\frac{H_{j}-{\hat{\mu }}_{x_{j}|q=i}\left(t\right)}{{\hat{\sigma }}_{x_{j}|q=i}\left(t\right)}\right).$$ (17)

Substituting Eq (17) into Eq (14), the reliability of the system is approximated by

$$R\left(t\right)\approx R_{e}\left(t\right)=\sum_{i=1}^{n-1}\mu_{i}^{\left(0,0,\ldots ,0\right)}\left(t\right)\cdot \left(\prod_{j=1}^{l}\Phi \left(\frac{H_{j}-{\hat{\mu }}_{x_{j}|q=i}\left(t\right)}{{\hat{\sigma }}_{x_{j}|q=i}\left(t\right)}\right)\right),$$ (18)

where ${\hat{\mu }}_{x_{j}|q=i}\left(t\right)$, ${\hat{\sigma }}_{x_{j}|q=i}\left(t\right)$ are calculated by Eq (15).

The accuracy of the approximation by FOSM relies on the normality assumption: the random variables *x*_*j*_(*t*)|*q*(*t*) = *i*, *i*∈1, 2, …, *n* − 1, *j* = 1, 2, ⋯, *l* are normally distributed with mean value $\mu_{x_{j}|q=i}\left(t\right)$ and standard deviation $\sigma_{x_{j}|q=i}\left(t\right)$. In practice, the assumption does not always hold. Therefore, we also present an estimation method for the lower bound of the system reliability, using Markov inequality.

According to Markov inequality [46], if *X* is a nonnegative random variable and *a*>0, then

$$\text{Pr}\left(X\geq a\right)\leq \frac{E\left(X\right)}{a}.$$ (19)

Using Eq (19), we obtain

$$\text{Pr}\left(x_{j}\left(t\right)\geq H_{j}|q=i\right)\leq \frac{E\left(x_{j}\left(t\right)|q=i\right)}{H_{j}},j\in \left\{1,2,\ldots ,l\right\},i\in \left\{1,2,\ldots ,n-1\right\}.$$ (20)

From Eqs (14) and (20), the lower bound of system reliability can, then, be derived:

$$\begin{array}{ll} R\left(t\right) & =\sum_{i=1}^{n-1}\text{Pr}\left(q\left(t\right)=i\right)\cdot \prod_{j=1}^{l}\text{Pr}\left(x_{j}\left(t\right)<H_{j}|q\left(t\right)=i\right)\\ & =\sum_{i=1}^{n-1}\text{Pr}\left(q\left(t\right)=i\right)\cdot \prod_{j=1}^{l}\left[1-\text{Pr}\left(x_{j}\left(t\right)\geq H_{j}|q\left(t\right)=i\right)\right]\\ & \geq \sum_{i=1}^{n-1}\text{Pr}\left(q\left(t\right)=i\right)\cdot \prod_{j=1}^{l}\left[1-\frac{E\left(x_{j}\left(t\right)|q\left(t\right)=i\right)}{H_{j}}\right]\\ & =\sum_{i=1}^{n-1}\mu_{i}^{\left(0,0,...,0\right)}\left(t\right)\cdot \prod_{j=1}^{l}\left[1-\frac{\mu_{i}^{\left(m^{*,j}\right)}\left(t\right)}{H_{j}}\right],\\ R_{l}\left(t\right) & =\sum_{i=1}^{n-1}\mu_{i}^{\left(0,0,...,0\right)}\left(t\right)\cdot \prod_{j=1}^{l}\left[1-\frac{\mu_{i}^{\left(m^{*,j}\right)}\left(t\right)}{H_{j}}\right], \end{array}$$ (21)

where m*^,*j*^ has the same meaning as in Eq (16).

## Results and discussion

### Case 1

#### System description

The first case study to demonstrate the developed framework is adapted from [20]. A MEMS device is subject to two dependent failure processes, i.e., soft failures caused by wear and debris from shock loads, and hard failures due to spring fracture caused by shock loads [20]. The soft failure is modeled by a continuous degradation process and the hard failure is modeled by a random shock process. Dependence exists among the two processes: the arrival of a shock brings an additional contribution to the degradation process. Failures occur whenever one of the following two events happens:

- the degradation process reaches its threshold, denoted by *H*;
- a shock whose magnitude exceeds a critical level, denoted by *D*, occurs.

Additional assumptions include:

1. The continuous degradation process follows an SDE.
  $$\;dx\left(t\right)=\mu_{\beta }dt+\sigma_{\beta }dw_{t},x\left(t\right)\in \mathbb{R},$$ (22)
  where *w*_*t*_ ∈ ℝ is a standard Wiener process, *μ*_*β*_, *σ*_*β*_ are constants and the initial degradation level at *t* = 0 is null.
2. The random shock process is a HPP with intensity *λ*.
3. The magnitudes of shock loads, denoted by *W*_*i*_, are i.i.d. random variables following a normal distribution, $W_{i}∼N\left(\mu_{W},\sigma_{W}^{2}\right)$.
4. The arrival of each shock brings a degradation increment *d*, which is a random variable following a normal distribution $d∼N(\mu_{d},\sigma_{d}^{2})$.

#### SHS formulation

**Fig 2:**
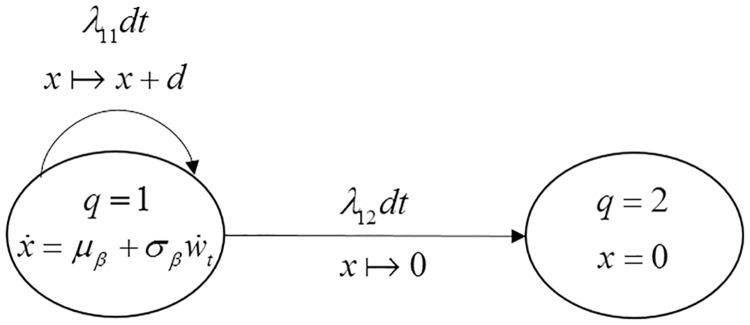
State-transition diagram of the SHS for case 1.

A SHS model is constructed in Fig 2 to describe the behavior of the system. The system has two health states, *q*(*t*)∈{1,2}. When *q*(*t*) = 1, the system is subject to the degradation process according to Eq (22). When *q*(*t*) = 2, the system fails due to hard failure and the degradation level is set to zero, i.e. *x*(*t*) = 0.

As shown in Fig 2, the initial health state of the system is state 1. The transition rates and reset maps of the SHS are defined as follows:

$$\begin{array}{l} \lambda_{11}\left(q\right):=\{\begin{array}{ll} \Phi \left(\frac{D-\mu_{W}}{\sigma_{W}}\right)\cdot \lambda \; & q=1,\\ 0 & q=2, \end{array}\\ \lambda_{12}\left(q\right):=\{\begin{array}{ll} \left(1-\Phi \left(\frac{D-\mu_{W}}{\sigma_{W}}\right)\right)\cdot \lambda & \;q=1,\\ 0 & \;q=2. \end{array} \end{array}$$ (23)

$$\begin{array}{l} \phi_{11}\left(q,x\right):=\left(1,x+d\right),\\ \phi_{12}\left(q,x\right):=\left(2,0\right). \end{array}$$ (24)

In Eq (24)), the reset map *ϕ*_11_(*q*,*x*) models the dependency in Assumption Eq (4).

#### Reliability analysis

We define the test functions $\psi_{i}^{\left(m\right)}\left(q,x\right),i\in \left\{1,2\right\},m\in \mathbb{R},$ to be:

$$\begin{array}{l} \psi_{1}^{\left(m\right)}\left(q,x\right)=\{\begin{array}{ll} x^{m} & q=1\\ 0 & q\neq 1, \end{array}\\ \psi_{2}^{\left(0\right)}\left(q,x\right)=\{\begin{array}{ll} 1 & q=2\\ 0 & q\neq 2. \end{array} \end{array}$$ (25)

Since *x*(*t*) = 0 when *q*(*t*) = 2, we only consider the 0-order conditional moment of the degradation at state 2, i.e. $\psi_{2}^{\left(0\right)}\left(q,x\right)$. By substituting Eqs (22), (23) and (24) into Eq (8), the extended generator of the SHS model is:

$$\begin{array}{ll} {\left(L\psi_{1}\right)}^{\left(m\right)}\left(q,x\right) & =\mu_{\beta }\frac{\partial \psi_{1}^{\left(m\right)}\left(q,x\right)}{\partial x}+\frac{1}{2}\sigma_{\beta }^{2}\frac{\partial^{2}\psi_{1}^{\left(m\right)}\left(q,x\right)}{\partial x^{2}}\\ & \;+\lambda_{11}\left(q\right){\left(\psi_{1}^{\left(1\right)}\left(q,x\right)+d\cdot \psi_{1}^{\left(0\right)}\left(q,x\right)\right)}^{\left(m\right)}\\ & \;-\left(\lambda_{11}\left(q\right)+\lambda_{12}\left(q\right)\right)\psi_{1}^{\left(m\right)}\left(q,x\right),\\ {\left(L\psi_{2}\right)}^{\left(0\right)}\left(q,x\right) & =\lambda_{12}\left(q\right)\cdot \psi_{1}^{\left(0\right)}\left(q,x\right). \end{array}$$ (26)

According to Eqs (7) and (26), the differential equations governing the conditional moments are:

$$\begin{array}{l} \frac{d}{dt}\mu_{1}^{\left(m\right)}\left(t\right)=\mu_{\beta }m\mu_{1}^{\left(m-1\right)}\left(t\right)+\frac{1}{2}\sigma_{\beta }^{2}m\left(m-1\right)\mu_{1}^{\left(m-2\right)}\left(t\right)\\ \;+\lambda_{11}\left(\sum_{k=0}^{m}\left(\begin{array}{c} m\\ k \end{array}\right)\mu_{1}^{\left(m-k\right)}\left(t\right)E\left(d^{k}\right)\right)-\left(\lambda_{11}+\lambda_{12}\right)\mu_{1}^{\left(m\right)}\left(t\right),\\ \frac{d}{dt}\mu_{2}^{\left(0\right)}\left(t\right)=\lambda_{12}\mu_{1}^{\left(0\right)}\left(t\right), \end{array}$$ (27)

where $E\left(d\right)=\mu_{d},E\left(d^{2}\right)=\mu_{d}^{2}+\sigma_{d}^{2}$. From Eq (27), we can obtain the following set of differential equations:

$$\left[\begin{array}{c} {\dot{\mu }}_{1}^{\left(0\right)}\\ {\dot{\mu }}_{1}^{\left(1\right)}\\ {\dot{\mu }}_{1}^{\left(2\right)} \end{array}\right]=\left[\begin{array}{ccc} -\lambda_{12} & 0 & 0\\ \mu_{\beta }+\lambda_{11}\mu_{d} & -\lambda_{12} & 0\\ \sigma_{\beta }^{2}+\lambda_{11}\left(\mu_{d}^{2}+\sigma_{d}^{2}\right) & 2\mu_{\beta }+2\lambda_{11}\mu_{d} & -\lambda_{12} \end{array}\right]\left[\begin{array}{c} \mu_{1}^{\left(0\right)}\\ \mu_{1}^{\left(1\right)}\\ \mu_{1}^{\left(2\right)} \end{array}\right].$$ (28)

Since the system must belong to one of the two health modes, it is obvious that

$$\mu_{1}^{\left(0\right)}\left(t\right)+\mu_{2}^{\left(0\right)}\left(t\right)=1.$$ (29)

From Eqs (18) and (21), the estimated system reliability *R*_*e*_(*t*) and the lower bound *R*_*l*_(*t*) are calculated by

$$R_{e}\left(t\right)=\mu_{1}^{\left(0\right)}\left(t\right)\cdot \Phi \left(\frac{H-\mu_{1}^{\left(1\right)}\left(t\right)/\mu_{1}^{\left(0\right)}\left(t\right)}{\sqrt{\mu_{1}^{\left(2\right)}\left(t\right)/\mu_{1}^{\left(0\right)}\left(t\right)+{\left(\mu_{1}^{\left(1\right)}\left(t\right)/\mu_{1}^{\left(0\right)}\left(t\right)\right)}^{2}}}\right),$$ (30)

$$R_{l}\left(t\right)=\mu_{1}^{\left(0\right)}\left(t\right)\cdot \left[1-\frac{\mu_{1}^{\left(1\right)}\left(t\right)}{H}\right],$$ (31)

where the conditional moments $\mu_{1}^{\left(0\right)}\left(t\right),\mu_{1}^{\left(1\right)}\left(t\right),\mu_{1}^{\left(2\right)}\left(t\right)$ are obtained by solving the differential equations in Eqs (28) and (29).

#### Numerical calculation

**Fig 3:**
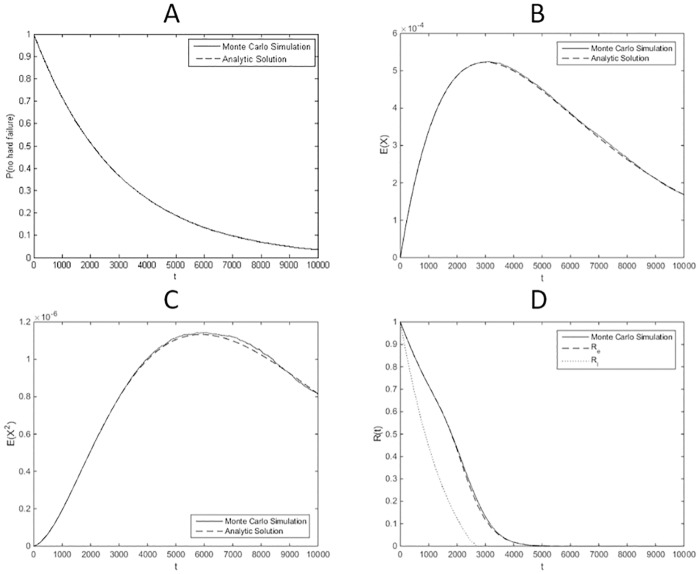
Comparison of results for case 1. (A) Comparison on the order 0 moment: $\text{Pr}\left\{q\neq 2\right\}=\mu_{1}^{\left(0\right)}\left(t\right)$. (B) Comparison on the order 1 moment: $E\left(x\left(t\right)\right)=\mu_{1}^{\left(1\right)}\left(t\right)+\mu_{2}^{\left(1\right)}\left(t\right)$. (C) Comparison on the order 2 moment: $E\left(x^{2}\left(t\right)\right)=\mu_{1}^{\left(2\right)}\left(t\right)+\mu_{2}^{\left(2\right)}\left(t\right)$. (D) Comparison on the estimated reliability and lower bound for reliability.

A numerical example is conducted with the parameters in Table 1, taken from [20]. To solve the differential equations in Eqs (28) and (29), the solver based on Runge Kutta method in Matlab R2013a is used. In [20], the original model is simulated using Monte Carlo methods to analyze system reliability. A comparison is made between the results obtained by the developed methods in this paper and those obtained by Monte Carlo simulation. The sample size of the Monte Carlo simulation is 10^4^. In Fig 3(A)–3(C), the moments with order 0, 1 and 2 are compared with simulation results. In Fig 3(D), the estimated reliability and its lower bound are compared to the results of Monte Carlo simulation.

**Table 1:** Parameter values for case 1.

Parameters	Value
*H*	0.00125*μm*^3^
*μ*_*β*_	8.4823×10^−9^*μm*^3^
*σ*_*β*_	6.0016×10^−10^*μm*^3^
*μ*_*d*_	1×10^−4^*μm*^3^
*σ*_*d*_	2×10^−5^*μm*^3^
*D*	1.5*GPa*
*λ*	5×10^−3^
*μ*_*W*_	1.2*GPa*
*σ*_*W*_	0.2*GPa*

The comparisons show that the moments are accurately predicted by the SHS model. The estimated reliability by FOSM is consistent with the result by Monte Carlo simulation. The estimated lower bound provides a relatively conservative reliability estimation. The running time of Monte Carlo simulation is 5788.2 times more than that of the developed SHS-based approach.

### Case 2

#### System description

The second case study to demonstrate the developed framework is adapted from [24]. A MEMS device is subject to two dependent failure processes, i.e., soft failures and hard failures. The soft failure is modeled by a continuous degradation process and the hard failure is modeled by a random shock process. Dependences between the two failure processes exist in the following two aspects: (1) the arrival of each shock brings a degradation increment; (2) the degradation rate increases when the system undergoes a series of shocks. The second type of dependence has been investigated based on four different shock models in [24]: extreme shock, *δ* shock, *m* shock, and run shock models. In this section, we apply our framework by considering the first dependence and the second dependence triggered by the extreme shock model. Failure occurs whenever one of the following two events happens:

- the degradation process reaches its threshold, denoted by *H*;
- a shock whose magnitude exceeds a critical level, denoted by *D*_1_, occurs.

Additional assumptions include:

1. Random shocks arrive according to a HPP with intensity *λ*.
2. The magnitudes of shock loads, denoted by *W*_*i*_, are i.i.d. random variables following a normal distribution $W_{i}∼N\left(\mu_{W},\sigma_{W}^{2}\right)$.
3. The arrival of a shock whose magnitude is less than *D*_0_ would bring a degradation increment *d*, which is a random variable following a normal distribution $d∼N(\mu_{d},\sigma_{d}^{2})$.
4. The arrival of a shock whose magnitude belongs to [*D*_0_, *D*_1_) would trigger the change of degradation rate. Let *J* denote the total number of arrival shocks when the trigger shock occurs, and *T*_*J*_ denote the time when the *J*th shock arrives. Then, the continuous degradation process is modeled by the following SDEs,
  $$\;dx\left(t\right)=\left\{ \begin{array}{l} \mu_{\beta_{1}}dt+\sigma_{\beta_{1}}dw_{t}\;for\;t<T_{J}\\ \mu_{\beta_{2}}dt+\sigma_{\beta_{2}}dw_{t}\;for\;t\geq T_{J} \end{array}\right.,x\left(t\right)\in \mathbb{R},$$ (32)
  where *w*_*t*_∈ℝ is a standard Wiener process, $\mu_{\beta_{1}},\mu_{\beta_{2}},\sigma_{\beta_{1}},\sigma_{\beta_{2}},\mu_{\beta_{2}}>\mu_{\beta_{1}}$ are constants, and the initial degradation level at *t* = 0 is null.

#### SHS formulation

**Fig 4:**
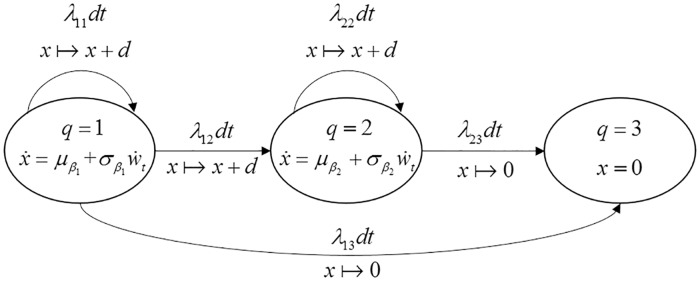
State-transition diagram for the SHS in case 2.

The SHS concerning this case is described by the state-transition diagram in Fig 4. The system has three health states, *q*(*t*)∈{1,2,3} When *q*(*t*) = 1, the system is subject to the degradation at a low-level degradation rate. By contrast, when *q*(*t*) = 2, the system degrades at a high-level degradation rate. System’s degradation under the first two health states evolves according to the following SDEs:

$$dx\left(t\right)=\left\{ \begin{array}{l} \mu_{\beta_{1}}dt+\sigma_{\beta_{1}}dw_{t}\;if\;q\left(t\right)=1\\ \mu_{\beta_{2}}dt+\sigma_{\beta_{2}}dw_{t}\;if\;q\left(t\right)=2 \end{array}\right.,x\left(t\right)\in \mathbb{R}.$$ (33)

When *q*(*t*) = 3, the system fails due to a hard failure and the degradation level is set to zero, i.e. *x*(*t*) = 0.

As shown in Fig 4, the initial health state of the system is state 1. The transition rates and reset maps of the SHS are defined as follows:

$$\begin{array}{l} \lambda_{11}\left(q\right):=\{\begin{array}{ll} P_{1}\cdot \lambda & q=1,\\ 0 & q\neq 1, \end{array}\\ \lambda_{12}\left(q\right):=\{\begin{array}{ll} P_{2}\cdot \lambda & q=1,\\ 0 & q\neq 1, \end{array}\\ \lambda_{13}\left(q\right):=\{\begin{array}{ll} P_{3}\cdot \lambda & q=1,\\ 0 & q\neq 1, \end{array}\\ \lambda_{22}\left(q\right):=\{\begin{array}{ll} \left(P_{1}\text{+}P_{2}\right)\cdot \lambda & q=2,\\ 0 & q\neq 2, \end{array}\\ \lambda_{23}\left(q\right):=\{\begin{array}{ll} P_{3}\cdot \lambda & q=2,\\ 0 & q\neq 2. \end{array} \end{array}$$ (34)

$$\begin{array}{l} \phi_{11}\left(q,x\right):=\left(1,x+d\right),\\ \phi_{12}\left(q,x\right):=\left(2,x+d\right),\\ \phi_{13}\left(q,x\right):=\left(3,0\right),\\ \phi_{22}\left(q,x\right):=\left(2,x+d\right),\\ \phi_{23}\left(q,x\right):=\left(3,0\right), \end{array}$$ (35)

where *P*_1_, *P*_2_, *P*_3_ are calculated by

$$P_{1}=\Phi \left(\frac{D_{\text{0}}-\mu_{W}}{\sigma_{W}}\right),P_{2}=\Phi \left(\frac{D_{1}-\mu_{W}}{\sigma_{W}}\right)-\Phi \left(\frac{D_{\text{0}}-\mu_{W}}{\sigma_{W}}\right),P_{3}=1-\Phi \left(\frac{D_{1}-\mu_{W}}{\sigma_{W}}\right).$$ (36)

In Eq (35), the reset maps *ϕ*_11_(*q*,*x*),*ϕ*_22_(*q*,*x*), model the dependency in assumption Eq (3) and the change of system degradation rate along with the transition from health state *q* = 1 to health state *q* = 2 model the dependency in assumption Eq (4).

#### Reliability analysis

We define the test functions $\psi_{i}^{\left(m\right)}\left(q,x\right),i\in \left\{1,2,3\right\},m\in \mathbb{R},$ to be:

$$\begin{array}{l} \psi_{1}^{\left(m\right)}\left(q,x\right)=\{\begin{array}{ll} x^{m} & q=1,\\ 0 & q\neq 1, \end{array}\\ \psi_{2}^{\left(m\right)}\left(q,x\right)=\{\begin{array}{ll} x^{m} & q=2,\\ 0 & q\neq 2, \end{array}\\ \psi_{3}^{\left(0\right)}\left(q,x\right)=\{\begin{array}{ll} 1 & q=3,\\ 0 & q\neq 3. \end{array} \end{array}$$ (37)

Since *x*(*t*) = 0 when *q*(*t*) = 3, we only consider the 0-order conditional moment of the degradation at state 3, i.e. $\psi_{3}^{\left(0\right)}\left(q,x\right)$. By substituting Eqs (33), (34) and (35) into Eq (8), the extended generator of the SHS is:

$$\begin{array}{l} {\left(L\psi_{1}\right)}^{\left(m\right)}\left(q,x\right)=\mu_{\beta_{1}}\frac{\partial \psi_{1}^{\left(m\right)}\left(q,x\right)}{\partial x}+\frac{1}{2}\sigma_{\beta_{1}}^{2}\frac{\partial^{2}\psi_{1}^{\left(m\right)}\left(q,x\right)}{\partial x^{2}}+\lambda_{11}{\left(\psi_{1}^{\left(1\right)}\left(q,x\right)+d\cdot \psi_{1}^{\left(0\right)}\left(q,x\right)\right)}^{\left(m\right)}\\ \;-\left(\lambda_{11}+\lambda_{12}+\lambda_{13}\right)\psi_{1}^{\left(m\right)}\left(q,x\right),\\ {\left(L\psi_{2}\right)}^{\left(m\right)}\left(q,x\right)=\mu_{\beta_{2}}\frac{\partial \psi_{2}^{\left(m\right)}\left(q,x\right)}{\partial x}+\frac{1}{2}\sigma_{\beta_{2}}^{2}\frac{\partial^{2}\psi_{2}^{\left(m\right)}\left(q,x\right)}{\partial x^{2}}+\lambda_{22}{\left(\psi_{2}^{\left(1\right)}\left(q,x\right)+d\cdot \psi_{2}^{\left(0\right)}\left(q,x\right)\right)}^{\left(m\right)}\\ \;+\lambda_{12}{\left(\psi_{1}^{\left(1\right)}\left(q,x\right)+d\cdot \psi_{1}^{\left(0\right)}\left(q,x\right)\right)}^{\left(m\right)}-\left(\lambda_{22}+\lambda_{23}\right)\psi_{2}^{\left(m\right)}\left(q,x\right),\\ {\left(L\psi_{3}\right)}^{\left(0\right)}\left(q,x\right)=\lambda_{13}\cdot \psi_{1}^{\left(0\right)}\left(q,x\right)+\lambda_{23}\cdot \psi_{2}^{\left(0\right)}\left(q,x\right). \end{array}$$ (38)

According to Eqs (7) and (38), the differential equations governing the conditional moments are:

$$\begin{array}{l} \frac{d}{dt}\mu_{1}^{\left(m\right)}\left(t\right)=\mu_{\beta_{1}}m\mu_{1}^{\left(m-1\right)}\left(t\right)+\frac{1}{2}\sigma_{\beta_{1}}^{2}m\left(m-1\right)\mu_{1}^{\left(m-2\right)}\left(t\right)\\ \;+\lambda_{11}\left(\sum_{k=0}^{m}\left(\begin{array}{c} m\\ k \end{array}\right)\mu_{1}^{\left(m-k\right)}\left(t\right)E\left(d^{k}\right)\right)-\left(\lambda_{11}+\lambda_{12}+\lambda_{13}\right)\mu_{1}^{\left(m\right)}\left(t\right),\\ \frac{d}{dt}\mu_{2}^{\left(m\right)}\left(t\right)=\mu_{\beta_{2}}m\mu_{2}^{\left(m-1\right)}\left(t\right)+\frac{1}{2}\sigma_{\beta_{2}}^{2}m\left(m-1\right)\mu_{2}^{\left(m-2\right)}\left(t\right)+\lambda_{22}\left(\sum_{k=0}^{m}\left(\begin{array}{c} m\\ k \end{array}\right)\mu_{2}^{\left(m-k\right)}\left(t\right)E\left(d^{k}\right)\right)\\ \;+\lambda_{12}\left(\sum_{k=0}^{m}\left(\begin{array}{c} m\\ k \end{array}\right)\mu_{1}^{\left(m-k\right)}\left(t\right)E\left(d^{k}\right)\right)-\left(\lambda_{22}+\lambda_{23}\right)\mu_{2}^{\left(m\right)}\left(t\right),\\ \frac{d}{dt}\mu_{3}^{\left(0\right)}\left(t\right)=\lambda_{13}\mu_{1}^{\left(0\right)}\left(t\right)+\lambda_{23}\mu_{2}^{\left(0\right)}\left(t\right), \end{array}$$ (39)

where $E\left(d\right)=\mu_{d},E\left(d^{2}\right)=\mu_{d}^{2}+\sigma_{d}^{2}$. From Eq (39), we can obtain the following set of differential equations:

$$\left[\begin{array}{l} \begin{array}{c} {\dot{\mu }}_{1}^{\left(0\right)}\\ {\dot{\mu }}_{2}^{\left(\text{0}\right)}\\ {\dot{\mu }}_{1}^{\left(\text{1}\right)} \end{array}\\ \begin{array}{c} {\dot{\mu }}_{2}^{\left(\text{1}\right)}\\ {\dot{\mu }}_{1}^{\left(\text{2}\right)}\\ {\dot{\mu }}_{2}^{\left(2\right)} \end{array} \end{array}\right]=\left[\begin{array}{cccccc} -\lambda_{12}-\lambda_{13} & \text{0} & \text{0} & \text{0} & \text{0} & \text{0}\\ \lambda_{12} & -\lambda_{23} & \text{0} & \text{0} & \text{0} & \text{0}\\ \mu_{\beta_{1}}+\lambda_{11}\mu_{d} & \text{0} & -\lambda_{12}-\lambda_{13} & \text{0} & \text{0} & \text{0}\\ \lambda_{12}\mu_{d} & \mu_{\beta_{2}}+\lambda_{22}\mu_{d} & \lambda_{12} & -\lambda_{23} & \text{0} & \text{0}\\ \sigma_{\beta_{1}}^{2}+\lambda_{11}\left(\mu_{d}^{2}+\sigma_{d}^{2}\right) & \text{0} & 2\mu_{\beta_{1}}+2\lambda_{11}\mu_{d} & \text{0} & -\lambda_{12}-\lambda_{13} & \text{0}\\ \lambda_{12}\left(\mu_{d}^{2}+\sigma_{d}^{2}\right) & \sigma_{\beta_{2}}^{2}+\lambda_{22}\left(\mu_{d}^{2}+\sigma_{d}^{2}\right) & 2\lambda_{12}\mu_{d} & 2\mu_{\beta_{2}}+2\lambda_{22}\mu_{d} & \lambda_{12} & -\lambda_{23} \end{array}\right]\left[\begin{array}{c} \mu_{1}^{\left(0\right)}\\ \mu_{2}^{\left(\text{0}\right)}\\ \mu_{1}^{\left(\text{1}\right)}\\ \mu_{2}^{\left(\text{1}\right)}\\ \mu_{1}^{\left(\text{2}\right)}\\ \mu_{2}^{\left(2\right)} \end{array}\right].$$ (40)

Since the system must belong to one of the three health modes, it is clear that

$$\mu_{1}^{\left(0\right)}\left(t\right)+\mu_{2}^{\left(0\right)}\left(t\right)+\mu_{3}^{\left(0\right)}\left(t\right)=1.$$ (41)

From Eqs (18) and (21), the estimated system reliability *R*_*e*_(*t*) and the lower boundary *R*_*l*_(*t*) are calculated by

$$R_{e}\left(t\right)=\sum_{i=1}^{2}\mu_{i}^{\left(0\right)}\left(t\right)\cdot \Phi \left(\frac{H-\mu_{i}^{\left(1\right)}\left(t\right)/\mu_{i}^{\left(0\right)}\left(t\right)}{\sqrt{\mu_{i}^{\left(2\right)}\left(t\right)/\mu_{i}^{\left(0\right)}\left(t\right)+{\left(\mu_{i}^{\left(1\right)}\left(t\right)/\mu_{i}^{\left(0\right)}\left(t\right)\right)}^{2}}}\right),$$ (42)

$$R_{l}\left(t\right)=\sum_{i=1}^{2}\mu_{i}^{\left(0\right)}\left(t\right)\cdot \left[1-\frac{\mu_{i}^{\left(1\right)}\left(t\right)}{H}\right].$$ (43)

where the conditional moments $\mu_{1}^{\left(0\right)}\left(t\right),\mu_{1}^{\left(1\right)}\left(t\right),\mu_{1}^{\left(2\right)}\left(t\right),\mu_{2}^{\left(0\right)}\left(t\right),\mu_{2}^{\left(1\right)}\left(t\right),\mu_{2}^{\left(2\right)}\left(t\right)$ are obtained by solving the differential Eqs (40) and (41).

#### Numerical calculation

**Fig 5:**
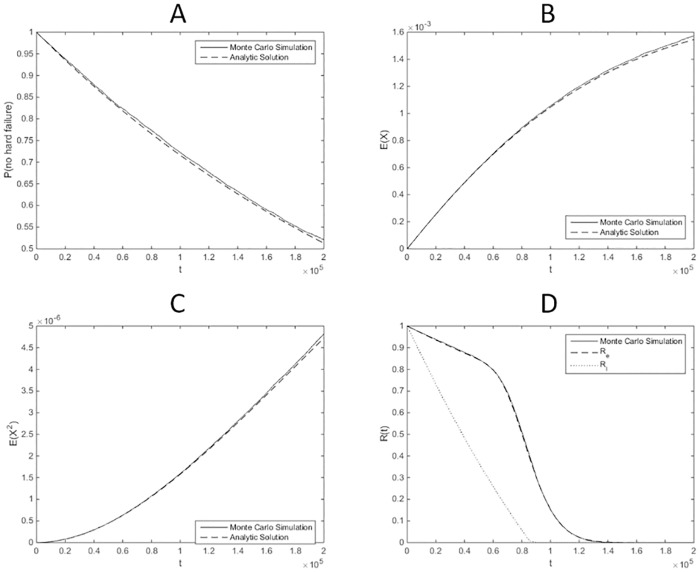
Comparison of results obtained by the SHS model and those by Monte Carlo simulation for case 2. (A) Comparison on the order 0 moment: $\text{Pr}\left\{q\neq 3\right\}=\mu_{1}^{\left(0\right)}\left(t\right)+\mu_{2}^{\left(0\right)}\left(t\right)$. (B) Comparison on the order 1 moment: $E\left(x\left(t\right)\right)=\mu_{1}^{\left(1\right)}\left(t\right)+\mu_{2}^{\left(1\right)}\left(t\right)+\mu_{3}^{\left(1\right)}\left(t\right)$. (C) Comparison on the order 2 moment: $E\left(x^{2}\left(t\right)\right)=\mu_{1}^{\left(2\right)}\left(t\right)+\mu_{2}^{\left(2\right)}\left(t\right)+\mu_{3}^{\left(2\right)}\left(t\right)$. (D) Comparison on the estimated reliability and lower bound for reliability.

A numerical example is conducted using the parameters in Table 2, taken from [24]. To solve the differential Eqs (40) and (41), the solver based on Runge Kutta method in Matlab R2013a is used. In [24], the original model is simulated by Monte Carlo to compute system reliability. A comparison is made between the results obtained by the developed methods in this paper and those obtained by Monte Carlo simulation. The sample size of the Monte Carlo simulation is 10^4^. In Fig 5(A)–5(C), the moments with order 0,1 and 2 are compared with the simulation results. In Fig 5(D), the estimated reliability and its lower bound are compared to the results of Monte Carlo simulation.

**Table 2:** Parameter values for case 2.

Parameters	Value
*H*	0.00125*μm*^3^
$\mu_{\beta_{1}}$	8.4823×10^−9^*μm*^3^
$\mu_{\beta_{2}}$	10.9646×10^−9^*μm*^3^
$\sigma_{\beta_{1}}$	6.0016×10^−10^*μm*^3^
$\sigma_{\beta_{2}}$	6.0846×10^−10^*μm*^3^
*μ*_*d*_	1×10^−4^*μm*^3^
*σ*_*d*_	2×10^−5^*μm*^3^
*D*_1_	1.5*GPa*
*D*_0_	1.2*GPa*
***λ***	5×10^−3^
*μ*_*W*_	1.2*GPa*
*σ*_*W*_	0.2*GPa*

The comparisons show that the moments are accurately predicted by the SHS model. The estimated reliability by FOSM is consistent with the result by Monte Carlo simulation. The estimated lower bound provides a relatively conservative reliability estimation. Besides, the running time of Monte Carlo simulation is 1203.8 times more than that of the developed SHS-based approach.

### Case 3

#### System descriptions

The third case study to demonstrate the developed framework is adapted from [25]. The failure behavior of bus tires is modeled by two dependent failure processes, i.e. soft failures caused by wear and hard failures of seven modes due to traumatic shocks. The soft failure is modeled by a continuous degradation process and the hard failure is modeled by a random shock process. Dependence exists among the two processes, that is, the probability that a traumatic shock occurs depends on the degradation level. Failures occur whenever one of the following two events happens:

- the degradation process reaches its threshold, denoted by *H*;
- a traumatic shock following a Cox process [47] with intensity *λ*(*x*) occurs.

Additional assumptions include:

1. The continuous degradation process is modeled by an SDE.
  $$\;dx\left(t\right)=\mu_{\beta }dt+\sigma_{\beta }dw_{t},x\left(t\right)\in \mathbb{R},$$ (44)
  where *w*_*t*_∈ℝ is a standard Wiener process, *μ*_*β*_,*σ*_*β*_ are constants and the initial degradation at *t* = 0 is denoted by *x*_0_.
2. Random shocks arrive according to a Cox process with intensity function *λ*(*x*) = *δ + αx*^*k*^,*k* ∈ℝ, where *δ* and *α* are constants. In this paper, we let *k* = 1. Then, the intensity function is determined by
  λ(x)=δ+αx. (45)

#### SHS formulation

**Fig 6:**
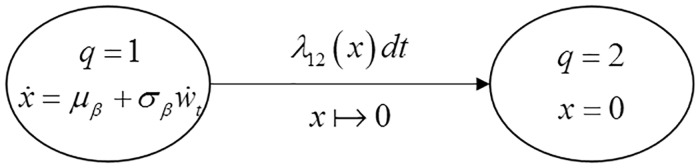
State-transition diagram for the SHS in case 3.

A SHS model is constructed in Fig 6 to describe the behavior of the system. The system has two health states, *q*(*t*)∈{1,2} When *q*(*t*) = 1, the system is subject to the degradation process and degrades according to Eq (44). When *q*(*t*) = 2, the system fails due to a hard failure, and the degradation level is set to zero, i.e. *x*(*t*) = 0.

As shown in Fig 6, the initial state of the system is state 1. The transition rates and reset maps of the SHS are defined as follows:

$$\lambda_{12}\left(q,x\right):=\{\begin{array}{ll} \delta +\alpha x & q=1,\\ 0 & q\neq 1, \end{array}$$ (46)

$$\phi_{12}\left(q,x\right):=\left(2,0\right).$$ (47)

#### Reliability analysis

We define the test functions $\psi_{i}^{\left(m\right)}\left(q,x\right),i\in \left\{1,2\right\},m\in \mathbb{R},$ to be:

$$\begin{array}{l} \psi_{1}^{\left(m\right)}\left(q,x\right)=\left\{ \begin{array}{l} x^{m}\;q=1,\\ 0\;q\neq 1, \end{array}\right.\\ \psi_{2}^{\left(0\right)}\left(q,x\right)=\left\{ \begin{array}{l} 1\;q=2,\\ 0\;q\neq 2. \end{array}\right. \end{array}$$ (48)

Since *x*(*t*) = 0 when *q*(*t*) = 2, we only consider the 0-order conditional moment of the degradation at state 2, i.e. $\psi_{2}^{\left(0\right)}\left(q,x\right)$. By substituting Eqs (44), (46) and (47) into Eq (8), the extended generator of the SHS is:

$$\begin{array}{l} {\left(L\psi_{1}\right)}^{\left(m\right)}\left(q,x\right)=\mu_{\beta }\frac{\partial \psi_{1}^{\left(m\right)}\left(q,x\right)}{\partial x}+\frac{1}{2}\sigma_{\beta }^{2}\frac{\partial^{2}\psi_{1}^{\left(m\right)}\left(q,x\right)}{\partial x^{2}}-\left(\delta +\alpha x\right)\cdot \psi_{1}^{\left(m\right)}\left(q,x\right),\\ {\left(L\psi_{2}\right)}^{\left(0\right)}\left(q,x\right)=\left(\delta +\alpha x\right)\cdot \psi_{1}^{\left(0\right)}\left(q,x\right). \end{array}$$ (49)

According to Eqs (7) and (49), the differential equations governing the conditional moments are:

$$\begin{array}{l} \frac{d}{dt}\mu_{1}^{\left(m\right)}\left(t\right)=\mu_{\beta }m\mu_{1}^{\left(m-1\right)}\left(t\right)+\frac{1}{2}\sigma_{\beta }^{2}m\left(m-1\right)\mu_{1}^{\left(m-2\right)}\left(t\right)-\delta \mu_{1}^{\left(m\right)}\left(t\right)-\alpha \mu_{1}^{\left(m+1\right)}\left(t\right),\\ \frac{d}{dt}\mu_{2}^{\left(0\right)}\left(t\right)=\delta \mu_{1}^{\left(0\right)}\left(t\right)+\alpha \mu_{1}^{\left(1\right)}\left(t\right). \end{array}$$ (50)

From Eq (50), we can obtain the following set of differential equations:

$$\left[\begin{array}{c} {\dot{\mu }}_{1}^{\left(0\right)}\\ {\dot{\mu }}_{1}^{\left(\text{1}\right)}\\ {\dot{\mu }}_{1}^{\left(\text{2}\right)} \end{array}\right]=\left[\begin{array}{ccc} -\delta & -\alpha & 0\\ \mu_{\beta } & -\delta & -\alpha \\ \sigma_{\beta }^{2} & 2\mu_{\beta } & -\delta \end{array}\right]\left[\begin{array}{c} \mu_{1}^{\left(0\right)}\\ \mu_{1}^{\left(\text{1}\right)}\\ \mu_{1}^{\left(\text{2}\right)} \end{array}\right]+\left[\begin{array}{c} 0\\ 0\\ -\alpha \end{array}\right]\cdot \mu_{1}^{\left(\text{3}\right)}.$$ (51)

Since the system must belong to one of the two health modes, it is obvious that

$$\mu_{1}^{\left(0\right)}\left(t\right)+\mu_{2}^{\left(0\right)}\left(t\right)=1.$$ (52)

Unlike the differential equations obtained in case 1 and case 2, the dynamics of $\mu_{1}^{\left(0\right)},\mu_{1}^{\left(1\right)},\mu_{1}^{\left(2\right)}$ in this case is related to a high-order conditional moment $\mu_{1}^{\left(3\right)}$, so that Eq (51) cannot be solved directly. To deal with this situation, an approximate truncation method is developed in [36] to provide an approximate function of the involved high-order conditional moments using the low-order conditional moments. Adopting the truncation method, we have the approximate function of $\mu_{1}^{\left(3\right)}$ in the following form:

$$\mu_{1}^{\left(3\right)}\approx \varphi \left(\mu_{1}^{\left(0\right)},\mu_{1}^{\left(1\right)},\mu_{1}^{\left(\text{2}\right)}\right)=\frac{\mu_{1}^{\left(0\right)}\cdot {\left(\mu_{1}^{\left(2\right)}\right)}^{3}}{{\left(\mu_{1}^{\left(1\right)}\right)}^{3}}.$$ (53)

Thus, Eq (51) is approximated by:

$$\left[\begin{array}{c} {\dot{\mu }}_{1}^{\left(0\right)}\\ {\dot{\mu }}_{1}^{\left(\text{1}\right)}\\ {\dot{\mu }}_{1}^{\left(\text{2}\right)} \end{array}\right]=\left[\begin{array}{ccc} -\delta & -\alpha & 0\\ \mu_{\beta } & -\delta & -\alpha \\ \sigma_{\beta }^{2} & 2\mu_{\beta } & -\delta \end{array}\right]\left[\begin{array}{c} \mu_{1}^{\left(0\right)}\\ \mu_{1}^{\left(\text{1}\right)}\\ \mu_{1}^{\left(\text{2}\right)} \end{array}\right]+\left[\begin{array}{c} 0\\ 0\\ -\alpha \end{array}\right]\cdot \frac{\mu_{1}^{\left(0\right)}\cdot {\left(\mu_{1}^{\left(2\right)}\right)}^{3}}{{\left(\mu_{1}^{\left(1\right)}\right)}^{3}}.$$ (54)

From Eqs (18) and (21), the estimated system reliability *R*_*e*_(*t*) and the lower bound *R*_*l*_(*t*) are calculated by

$$R_{e}\left(t\right)=\mu_{1}^{\left(0\right)}\left(t\right)\cdot \Phi \left(\frac{H-\mu_{1}^{\left(1\right)}\left(t\right)/\mu_{1}^{\left(0\right)}\left(t\right)}{\sqrt{\mu_{1}^{\left(2\right)}\left(t\right)/\mu_{1}^{\left(0\right)}\left(t\right)+{\left(\mu_{1}^{\left(1\right)}\left(t\right)/\mu_{1}^{\left(0\right)}\left(t\right)\right)}^{2}}}\right),$$ (55)

$$R_{l}\left(t\right)=\mu_{1}^{\left(0\right)}\left(t\right)\cdot \left[1-\frac{\mu_{1}^{\left(1\right)}\left(t\right)}{H}\right].$$ (56)

where the conditional moments $\mu_{1}^{\left(0\right)}\left(t\right),\mu_{1}^{\left(1\right)}\left(t\right),\mu_{1}^{\left(2\right)}\left(t\right)$ are obtained by solving the differential Eqs (52) and (54).

#### Numerical calculation

**Fig 7:**
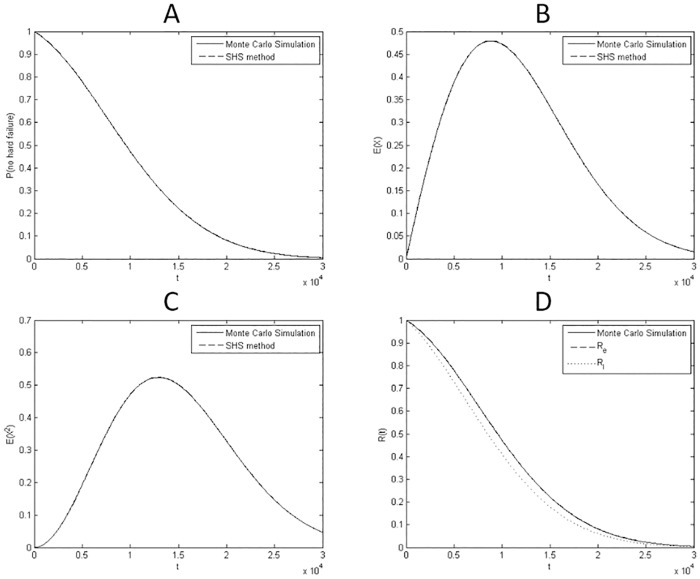
Comparison of results obtained by the SHS model and those by analytic expressions for case 3. (A) Comparison on the order 0 moment: $\text{Pr}\left\{q\neq 2\right\}=\mu_{1}^{\left(0\right)}\left(t\right)$. (B) Comparison on the order 1 moment: $E\left(x\left(t\right)\right)=\mu_{1}^{\left(1\right)}\left(t\right)+\mu_{2}^{\left(1\right)}\left(t\right)$. (C) Comparison on the order 2 moment: $E\left(x^{2}\left(t\right)\right)=\mu_{1}^{\left(2\right)}\left(t\right)+\mu_{2}^{\left(2\right)}\left(t\right)$. (D) Comparison on the estimated reliability and lower bound for reliability.

A numerical example is conducted using the parameters in Table 3, assumed arbitrarily by hypothesis for the purpose of illustration. To solve the differential equations in Eqs (52) and (54), the solver based on Runge Kutta method in Matlab 2013a is used. On the other hand, based on the properties of Cox process, the conditional moments can also be written as Eqs (57) and (58) below:

$$\begin{array}{l} \mu_{1}^{\left(0\right)}\left(t\right)=P\left\{q=1\right\}\\ \;=E\text{exp}\left(-\int_{\text{0}}^{t}\lambda \left(x\left(\tau \right)\right)d\tau \right)\\ \;=E\text{exp}\left(-\int_{\text{0}}^{t}\left(\delta +\alpha x\left(\tau \right)\right)d\tau \right),\\ \mu_{1}^{\left(1\right)}\left(t\right)=E\left(x|q=1\right)\cdot P\left\{q=1\right\}\\ \;=\left(x_{0}+\mu_{\beta }t\right)\cdot E\text{exp}\left(-\int_{\text{0}}^{t}\left(\delta +\alpha x\left(\tau \right)\right)d\tau \right),\\ \mu_{1}^{\left(2\right)}\left(t\right)=E\left(x^{2}|q=1\right)\cdot P\left\{q=1\right\}\\ \;=\left[{\left(x_{0}+\mu_{\beta }t\right)}^{2}+\sigma_{\beta }^{2}t\right]\cdot E\text{exp}\left(-\int_{\text{0}}^{t}\left(\delta +\alpha x\left(\tau \right)\right)d\tau \right). \end{array}$$ (57)

$$\begin{array}{l} R\left(t\right)=P\left\{x<H|q=1\right\}\cdot P\left\{q=1\right\}\\ \;=\Phi \left(\frac{H-\left(x_{0}+\mu_{\beta }t\right)}{\sigma_{\beta }\sqrt{t}}\right)\cdot E\text{exp}\left(-\int_{\text{0}}^{t}\left(\delta +\alpha x\left(\tau \right)\right)d\tau \right), \end{array}$$ (58)

where *x*_0_ denotes the initial degradation magnitude and Φ(⋅) represents the standard normal distribution function. A comparison is made between the results obtained by the developed methods in this paper and those obtained by solving Eqs (57) and (58) through Monte Carlo simulation. The sample size of the Monte Carlo simulation is 10^3^. In Fig 7(A)–7(C), the moments with order 0,1 and 2 are compared with those obtained by Eq (57). In Fig 7(D), the estimated reliability and its lower bound are compared to the results calculated by Eq (58). The comparisons show that the moments and system reliability are accurately predicted by the SHS model.

**Table 3:** Parameter values for case 3.

Parameters	Value
*H*	7.5*μm*^3^
*μ*_*β*_	1×10^−4^ *μm*^3^
*σ*_*β*_	1×10^−5^ *μm*^3^
*δ*	2.5 × 10^−5^
*α*	1×10^−4^
*x*_0_	1×10^−4^ *μm*^3^

## Conclusions

In this paper, a SHS-based modeling framework is developed for the reliability modeling and analysis of dependent failure processes, where degradation processes and random shock processes compete to cause system failure and dependencies exist among these processes. In the developed model, the degradation process is modeled by SDEs and the shock process is characterized by transitions among the system health states. The dependencies among the two processes are modeled within the structure of the SHS model by the reset map, transition rates etc. The conditional moments for the state variables in the developed SHS model are calculated by deriving and solving a set of differential equations based on Dynkin’s formula. Using these conditional moments, a reliability analysis method is developed to estimate the system reliability and its lower bound. Three case studies are conducted to demonstrate the developed methods. Comparisons to Monte Carlo simulations show that the developed method can achieve accurate reliability analysis results, while requiring much less computations than Monte Carlo simulations.

To apply the developed model in practice, the parameter values, such as the parameters in the SDEs that model the degradation processes, the transition rates that model the random shock processes, and the parameters in the reset maps that describe the dependency, need to be set based on historical data or expert judgments. Epistemic uncertainty might present when setting values for the parameters. Another source of epistemic (model) uncertainty is derived from the assumptions made for the present model. Treatment and calculation of epistemic uncertainty is an interesting problem that deserves further investigations.
